# Evaluating the effect of mobile applications “My A:Care” and “Smart Coach” on adherence to lipid-lowering treatment in patients with dyslipidemia: a prospective, randomized, open-label clinical study

**DOI:** 10.3389/fdgth.2025.1502990

**Published:** 2025-07-07

**Authors:** Chatlert Pongchaiyakul, Stefan Driessen

**Affiliations:** ^1^ Division of Endocrinology and Metabolism, Department of Medicine, Faculty of Medicine, Khon Kaen University, Khon Kaen, Thailand; ^2^ Established Pharmaceuticals Division, Global Biometrics, Abbott Healthcare Products B.V, Weesp, Netherlands

**Keywords:** dyslipidemia, medication adherence, medication non-adherence, mobile applications, behavioral interventions, lipid-lowering therapies

## Abstract

**Background:**

Dyslipidemia, a key modifiable risk factor for cardiovascular diseases, is managed using lipid-lowering therapies. Medication adherence for dyslipidemia treatment is poor across the globe, impacting treatment effectiveness. This highlights the need for scalable strategies, such as mobile app-based behavioral interventions, to enhance adherence to lipid-lowering therapies.

**Objective:**

The study assesses the impact of “My A:Care” and “My A:Care Smart Coach” mobile interventions on adherence to dyslipidemia treatment.

**Methods:**

This proof-of-concept, open-label, single-center study randomized 150 patients with suboptimal adherence to dyslipidemia treatment into three groups (1:1:1): My A:Care, My A:Care Smart Coach (intervention), and a no-app control group. Participants were monitored over 12 weeks. The primary objective was to assess changes in medication adherence, with secondary outcomes including changes in lipid parameters and beliefs about lipid-lowering medications. The study also explored the association between adherence and app engagement.

**Results:**

At week 12, the Medication Adherence Report with Visual Analog Scale (MARS-5VA) Part 1 scores were modestly, but significantly lower in the control group compared to the intervention groups: Mean (SD); No-App: −0.3 (0.9), Smart Coach: 0.0 (0.7) [*p* = 0.035], My A:Care-All: 0.0 (0.7) [*p* = 0.056]. Compared to the control, the intervention groups also showed greater improvements in non-HDL-C levels [% change (SE): My A:Care-All: −5.5% (3.2), Smart Coach: −4.3% (3.7), No-App: −1.8% (3.7)], along with favorable trends in TC, LDL-C, and HDL-C.

**Conclusion:**

This proof-of-concept study suggests that the My A:Care and Smart Coach apps may positively impact adherence to lipid-lowering therapy in patients with dyslipidemia. The positive adherence outcomes and potential benefits in lipid control indicate promising early signals that warrant further investigation in larger, confirmatory studies.

**Clinical Trial Registration:**

NCT05370703.

## Graphical Abstract

**Figure:**
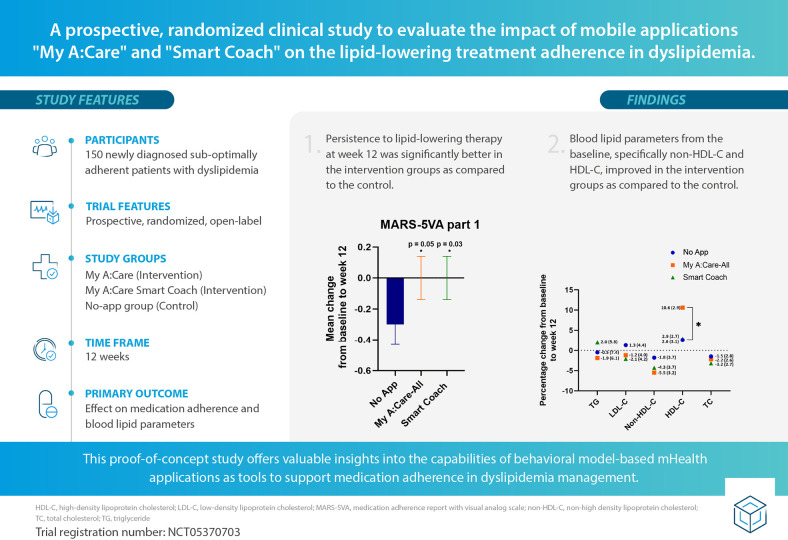

## 1 Introduction

Dyslipidemia, a key risk factor for cardiovascular diseases (CVD), is an important public health challenge. Dyslipidemia is defined as abnormal levels of low-density lipoprotein cholesterol (LDL-C), triglyceride-rich lipoproteins (TRLs), and high-density lipoprotein cholesterol (HDL-C) (1, 2). According to the World Health Organization (WHO), globally, about 39% of people over 25 years of age have higher than the recommended levels (>190 mg/dl) of total blood cholesterol, which is responsible for 4.4 million deaths each year—approximately one-third of all CVD-related mortality (3, 4).

Lipid-lowering therapy, in addition to dietary and lifestyle modifications, is pivotal for CVD prevention and management (5). Treatment with anti-lipidemic agents is reported to reduce the CVD risk by about 20%–25% (2, 6–8). Nevertheless, adherence to lipid-lowering agents in the real world is suboptimal. As with other chronic conditions, adherence to lipid-lowering therapies remains suboptimal, with average adherence rates estimated at approximately 50%. Persistence is particularly poor, with a substantial proportion of patients discontinuing therapy within the first year of initiation. Recent studies have reported low persistence rates—around 64%—especially among individuals without clinical symptoms or a documented history of CVD (9–11). Patients with lower adherence to lipid-lowering treatment are at significantly higher risk of CVD events compared to those who are more adherent (12, 13). These findings call for better and more impactful interventions to improve adherence to anti-lipidemic agents.

Suboptimal adherence to lipid-lowering medications is commonly attributed to forgetfulness, lack of information, fear of adverse effects, distrust in medications, poor patient-practitioner communication, and various socioeconomic factors. Interventions addressing these issues and helping to bring positive behavioral change, as well as a shift in attitude towards medications, are essential to enhance adherence (10). Moreover, such interventions need to be scalable and economical due to the change in the landscape of atherogenic lipid profiles with a marked shift in its epicenter from high to low-middle-income countries (14).

The widespread adoption of mobile phones, coupled with technological advancements, has fueled the growth of mobile health (mHealth) interventions that are practical, cost-effective, and capable of delivering personalized care (15). Numerous studies have reported positive outcomes from mHealth applications aimed at improving medication adherence across various chronic conditions (16–18). The existing mHealth interventions for CVD prevention typically support medication reminders, self-monitoring, lifestyle changes, health education, and promote behavior change using text, voice, or picture messages (19–21). A Cochrane review of 14 trials provided modest yet encouraging evidence for the impact of mHealth interventions in enhancing medication adherence (15). However, currently used mHealth applications make limited use of behavior change techniques and have not fully benefited from the advances in the field of health behavior change (22). Additionally, most of them lack involvement of healthcare professionals during development and have a limited evidence base (23). This highlights the need for further studies to develop and evaluate apps grounded in theory-based behavior change strategies, beyond simple reminder functions, to improve medication adherence.

This paper describes the findings from a proof-of-concept study to explore the effectiveness of two mHealth applications, “My A:Care” and “My A:Care Smart Coach”, in improving adherence to medication for dyslipidemia treatment by encouraging self-care among patients. The mobile applications are based on established behavioral methods and offer pill reminders, deliver health insights, motivational messages, challenges, and virtual rewards to encourage positive changes in patient behavior. My A:Care Smart Coach is an advanced version of the My A:Care application. The major difference is the use of SPUR (Social, Psychological, Usage, Rational) framework-enabled tailored treatment plans and messaging to patients that target patient-specific barriers to medication adherence.

## 2 Methods

### 2.1 Study design and objectives

This was a prospective, randomized, open-label, single-center clinical trial designed to evaluate the effectiveness of two behavioral mHealth applications—“My A:Care” and “My A:Care Smart Coach” (hereafter referred to as Smart Coach)—compared to standard of care (No App group) in patients with suboptimal adherence to dyslipidemia treatment. These novel mHealth applications are designed to address medication non-adherence using established behavioral methods (24–26). The trial was sponsored by Abbott Established Pharmaceuticals Division, Basel, Switzerland.

### 2.2 Description of the mobile health interventions

#### 2.2.1 My A:Care application

It is a novel mHealth application designed to address medication non-adherence using established behavioral methods. The application can be recommended to patients by healthcare professionals to encourage and monitor adherence. My A:Care associates adherence with self-care and helps patients take small, manageable steps while rewarding positive actions to facilitate lasting behavioral changes. The application provides motivational messages/challenges, health insights, and pill reminders to encourage adherence (24). Further details on the application are described in Supplementary Methods S1.1 and Supplementary Table 1.

#### 2.2.2 My A:Care Smart Coach

Smart Coach is an advanced version of the My A:Care application running on the same core engine with the same app interface. It differs substantially from the latter by delivering SPUR (Social, Psychological, Usage, Rational) framework-enabled patient profiling in combination with clinical and sociodemographic information using the d.Tells^TM^ algorithm to deliver tailored treatment plans (25, 26). Further details on the application and SPUR questionnaire are described in Supplementary Methods S1.2.

### 2.3 Outcome measures and assessment tools

#### 2.3.1 MARS-5VA (part 1 & 2)

Medication adherence was measured using the Medication Adherence Report with Visual Analog Scale (MARS-5VA) questionnaire. This questionnaire comprised 2 parts: a 5-item Medication Adherence Report Scale (MARS-5) as part 1 and two Visual Analogue Scales (VAS) as part 2. While MARS-5VA part 1 assesses non-adherence to any medication, MARS-5VA part 2 specifically evaluates adherence to lipid-lowering therapy. MARS-5 is a clinically validated and reliable five-item self-report questionnaire that captures both intentional and unintentional non-adherence with a minimal social desirability bias (27). The score in the MARS-5VA part 1 ranges from 5 to 25 for lower to higher adherence, respectively. Responses to the scale are non-dichotomous and provide a nuanced differentiation of the adherence behavior to allow categorizing respondents into multiple groups as per their position along the non-adherence continuum (28). Alternately, VAS is a single-item, simple-to-administer adherence scale utilizing the patient's recall to estimate the number of doses taken in a specified period. It has a 10-point response scale with values ranging from 0% (none used) to 100% (all used) (29).

#### 2.3.2 BMQ, PSM-5, and MAIN scores

Patients' general perception towards medications was evaluated using a combination of the twelve-item Beliefs about Medicines Questionnaire-General 12 (BMQ-General-12) (30) and a five-item Perceived Sensitivity to Medicines Scale-5 (PSM-5) questionnaire (31). The BMQ-General-12 score ranges from 4 to 20 for each subscale (overuse, harm, and benefit), with a high score indicating a more positive perception of medicines. Alternatively, PSM-5 score ranges from 5 to 25, with high scores correlating with higher perceived sensitivity to potential adverse effects of medicines.

To appraise patients' perceptions specific to lipid-lowering therapy, the Beliefs about Medicines Questionnaire—Specific (BMQ-S11-Plural) was employed (30). The BMQ-S11 Plural questionnaire has two subsets—BMQ-Specific-Necessity and BMQ-Specific-Concern. The Medicines Adherence and Information Needs (MAIN) Screen was used to determine the barriers and support needed for the participant's medicine adherence. Various barriers and facilitators of medication adherence were examined, for example, difficulty in using the medicine, support required for using the medicine, information, and advice available about the medicine, ease in availability of medicine, and necessity-concern beliefs.

### 2.4 Study procedures, inclusion, and exclusion criteria

#### 2.4.1 Inclusion and exclusion criteria

Participants aged 18–75 years with newly diagnosed dyslipidemia, initiated on stable lipid-lowering therapy between 1 and 9 months before the study start date, were included. A total of 150 patients from this primary prevention cohort were enrolled. Screening was conducted at Visit 1 using the MARS-5VA (Part 1), with a score <22 indicating suboptimal adherence and eligibility for inclusion. Key inclusion criteria included ownership of an Android or iOS smartphone and the ability and willingness to use it. Major exclusion criteria were a recent history (within 2 months) of myocardial infarction, stroke, or unstable angina; recent hospitalization for cardiovascular conditions; need for changes in lipid-lowering therapy; adverse effects requiring therapy modification or discontinuation; use of injectable lipid-lowering agents; or concurrent use of other adherence-related mHealth applications.

The study was approved by the Human Research Ethics Board of the Faculty of Medicine, Khon Kaen University (Reference no. HE651047, 1 April 2022). Written informed consent was obtained from all participants prior to their enrollment in the study.

#### 2.4.2 Study initiation

Trial participants were randomized 1:1:1 (50 each) into three groups, My A:Care and Smart Coach (intervention) and No App (control) using variable block sizes of 3, 6, or 9. The final block size was adjusted to ensure balanced allocation at the target sample size (*n* = 150). To obscure the sequence, the final assignment was moved to the first position, creating unbalanced blocks at both ends. An independent consultant generated the sequence and prepared sealed, opaque, sequentially numbered envelopes. Investigators assigned interventions by opening envelopes in order. Tamper-Resistant Envelope Allocation Technique (TREAT) and a random number chain check were employed to prevent tampering and ensure sequence integrity (32).

As this was an exploratory study, the sample size was not based on a formal sample size calculation. Participants' baseline characteristics, medical history, details of the lipid-lowering agent, comorbidities, and other concomitant treatments being administered were documented at visit 1. Patients allocated to the intervention groups were assisted in downloading the respective mobile application and received training on application usage. Patients assigned to the Smart Coach group were administered the in-app SPUR questionnaire for patient profiling (Supplementary Methods S1.2).

#### 2.4.3 Study duration and follow-up

The study duration was 3 months, with participants followed up at 12 weeks (±7 days) during Visit 2. Lipid panel data were collected at both baseline (Visit 1) and follow-up. Concomitant medications and any adverse drug reactions related to Abbott products were recorded. Mobile application usage data, including individual user summaries, were retrieved and assessed in pseudonymized form to evaluate interaction with the two applications.

### 2.5 Study endpoints and variations

The primary endpoint was the change from baseline in MARS-5VA (Parts 1 & 2) scores at week 12. Secondary endpoints included: (1) changes in lipid parameters—total cholesterol (TC), triglycerides (TG), LDL-C, HDL-C, and non-HDL-C; and (2) changes in beliefs about medications, both general (BMQ General-12 and PSM-5) and specific to lipid-lowering therapy (BMQ-S11-Plural). The exploratory endpoint assessed correlations between app engagement and changes in adherence, based on in-app features such as the cube (motivational messages/challenges) and battery (pill reminder) status (Supplementary Figure S1).

**Figure 1:**
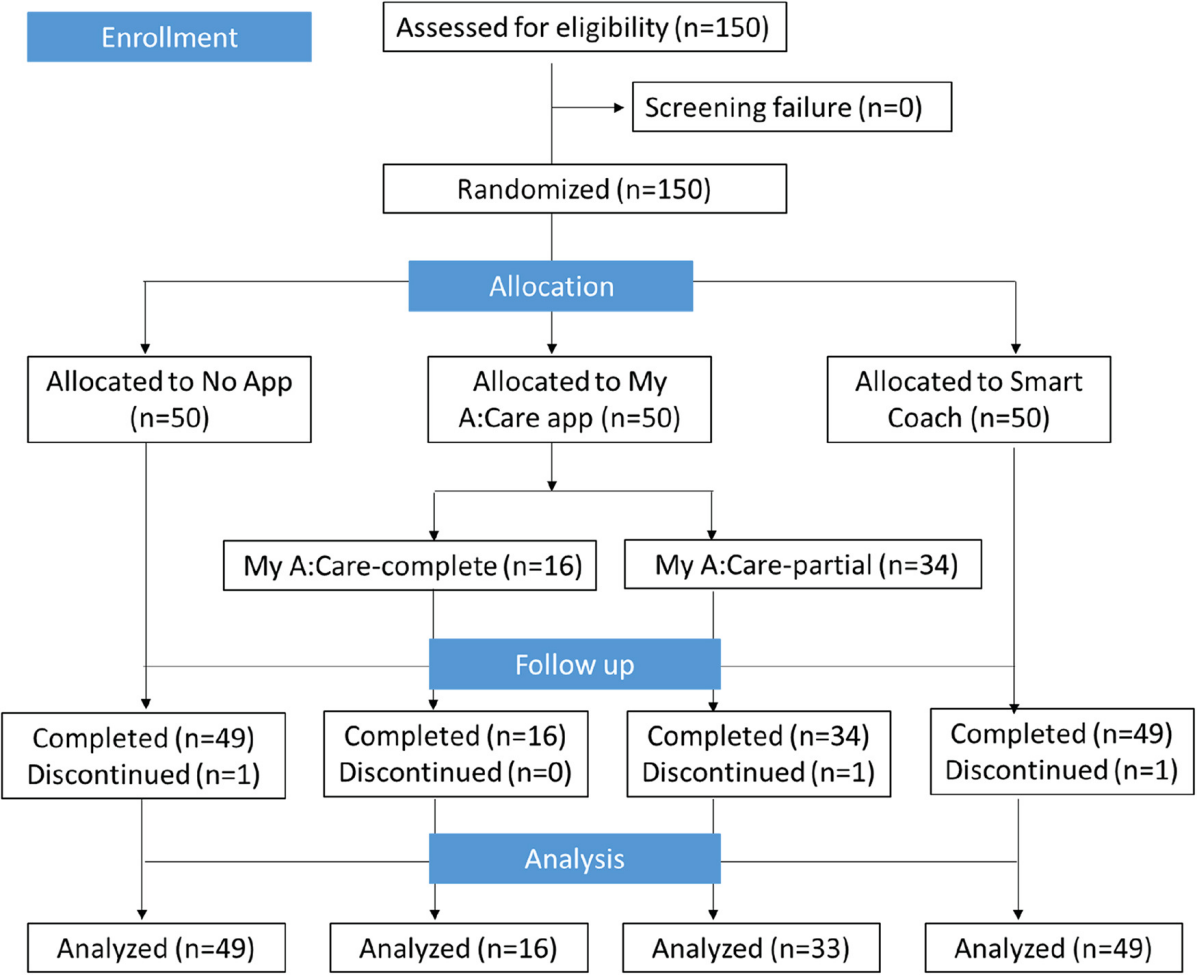
Study flow-chart.

Of the 150 enrolled patients, 147 completed the study; one patient from each group withdrew consent after Visit 1. In the My A:Care group, a technical glitch prevented app notifications for motivational messages/challenges in all but 16 participants. Consequently, for analysis, the My A:Care group was divided into two subgroups: My A:Care-Partial (*N* = 33), affected by the issue, and My A:Care-Complete (*N* = 16), who received the intervention as intended (Figure 1).

### 2.6 Statistical analysis

Effectiveness for the primary and secondary objectives was assessed using analysis of covariance (ANCOVA), adjusting for baseline values to account for group differences. Pairwise comparisons were conducted between the control and each intervention group, as well as between the two intervention groups. Unless stated otherwise, statistical significance was set at *p* < 0.05 (two-sided). Sensitivity analyses comparing changes from baseline to week 12 were performed using Wilcoxon's Rank-Sum test.

Changes in MARS-5VA part 1&2 scores from baseline to week 12 were correlated with exploratory variables using Pearson's correlation coefficient within the two intervention groups. To evaluate differences in user engagement patterns, all exploratory variables were compared between the two intervention groups using Wilcoxon's Rank-Sum test. Missing app usage data—whether due to disengagement, connectivity issues, or delayed transmission—was recorded as “red cube” (for missing cube status) or assigned a value of “0” (for missing battery status).

## 3 Results

### 3.1 Study population and demographics

The mean (SD) age of participants was 55.7 (9.6) years, with 59.2% aged between 49 and 64 years; 70% were female, and all but one (White) were of Asian ethnicity (Table 1). Most participants had at least one comorbidity in addition to dyslipidemia: No App (73.5%), Smart Coach (75.5%), My A:Care-Complete (100%), and My A:Care-All (75.5%). Type 2 diabetes and hypertension were the most common comorbidities. All patients were on multiple medications. Atorvastatin calcium and simvastatin were the most frequently used lipid-lowering agents, with generally comparable distribution across groups, except for a lower use of atorvastatin in the My A:Care-Complete group (37.5% vs. 51.0–55.1%) and lower use of simvastatin in the Smart Coach group (24.5% vs. 30.6–37.5%).

**Table 1:** Study demographics.

Participant characteristics	All subjects (*N* = 147)	No app (*N* = 49)	My A:Care-complete (*N* = 16)	My A:Care-All (*N* = 49)	Smart coach (*N* = 49)
Age in years					
Mean (SD)	55.7 (9.6)	55.5 (11.6)	56.6 (10.9)	57.3 (9.5)	54.1 (7.2)
Age category (years)					
≥18–34 n(%)	6 (4.1)	4 (8.2)	1 (6.3)	2 (4.1)	0
>34–49 n(%)	29 (19.7)	9 (18.4)	1 (6.3)	5 (10.2)	15 (30.6)
>49–64 n(%)	87 (59.2)	23 (46.9)	11 (68.8)	33 (67.3)	31 (63.3)
>64–75 n(%)	24 (16.3)	12 (24.5)	3 (18.8)	9 (18.4)	3 (6.1)
Gender					
Male n(%)	45 (30.6)	16 (32.7)	3 (18.8)	13 (26.5)	16 (32.7)
Female n(%)	102 (69.4)	33 (67.3)	13 (81.3)	36 (73.5)	33 (67.3)
Race					
White n(%)	1 (0.7)	0	0	0	1 (2.0)
Asian n(%)	146 (99.3)	49 (100.0)	16 (100.0)	49 (100.0)	48 (98.0)
Black n(%)	0	0	0	0	0
Other n(%)	0	0	0	0	0
Comorbidity		73.5	100	75.5	75.5
T2D %		20.4	56.3	36.7	24.5
Hypertension %		24.5	50	32.7	16.3

SD, standard deviation; T2D, type 2 diabetes.

Age (years) is calculated relative to screening.

### 3.2 Primary outcome: effectiveness of interventions on adherence to lipid-lowering therapy

**Figure 2:**
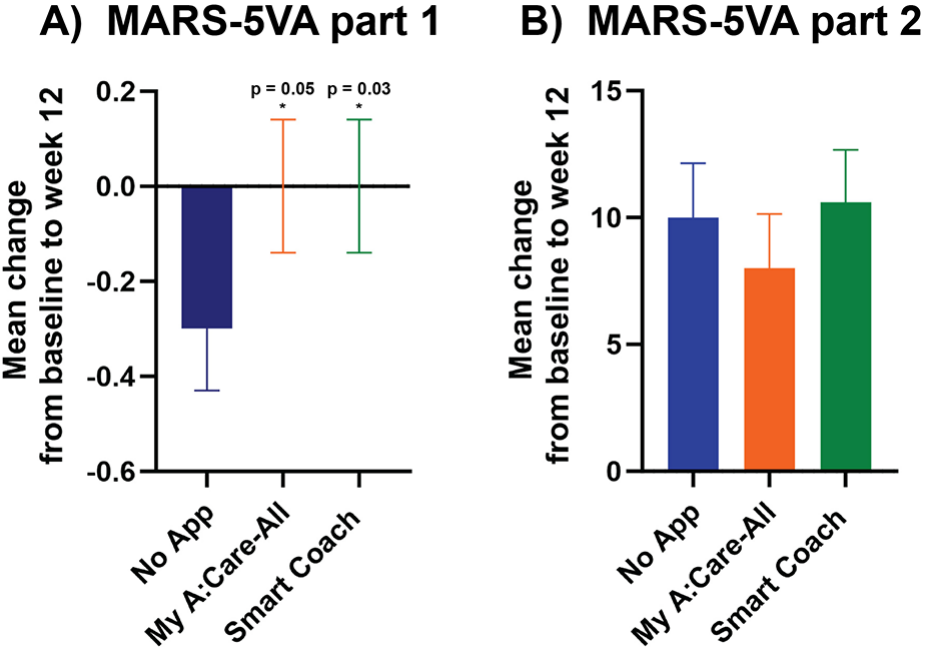
Persistence to medications was higher in intervention groups as compared to the control. **(A)** The mean changes in scores for MARS-5VA part 1 were significantly better in Smart Coach (ANCOVA analysis, *p* = 0.035) and borderline significant in My A:Care-All (ANCOVA analysis, *p* = 0.056) group when compared to the control. **(B)** The mean changes in the MARS-5VA part 2 score were better in My A:Care-All group but were not significant. *p* < 0.05. The error bars represent standard error.

Adherence to lipid-lowering therapy was assessed using the MARS-5VA (parts 1 & 2) questionnaire. From baseline to week 12, the mean change in MARS-5VA part 1 scores was significantly greater in the Smart Coach group compared to the No App group [mean (SD): 0.0 (0.7) vs. −0.3 (0.9); *p* = 0.035], and borderline significant for the My A:Care-All group vs. No App [0.0 (0.7) vs. −0.3 (0.9); *p* = 0.056] (Figure 2A).

The mean change in the MARS-5VA part 2 score at week 12 was not significantly different among the three groups [mean (SD): Smart Coach 10.6 (14.5); My A:Care-All 8.0 (15.0); No App 10.0 (14.6)] (Figure 2B).

### 3.3 Secondary outcome: effectiveness of interventions on serum lipid levels

**Figure 3:**
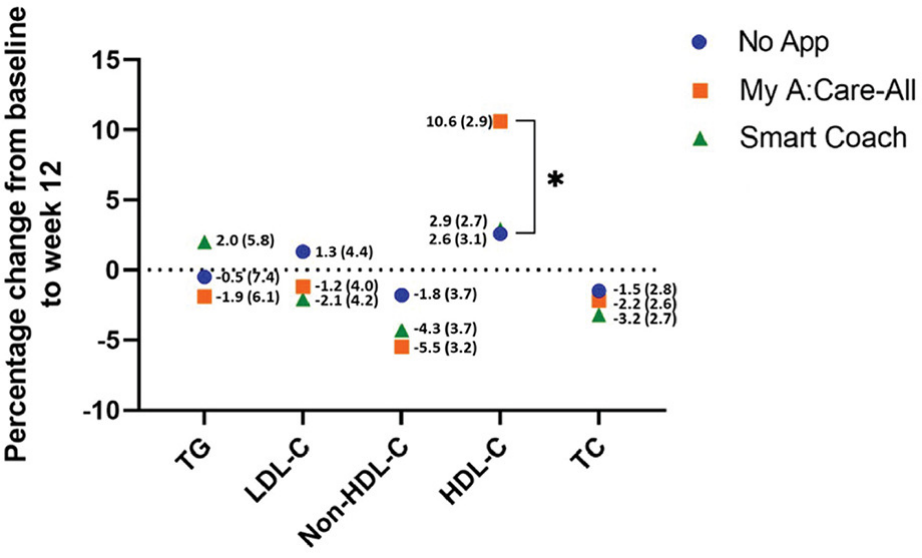
Lipid parameters improved in interventions as compared to the control. The superimposed scatter plot depicts the mean percentage change [mean% change (SE)] in various lipid parameters from baseline to the end of the study (week 12) among control, My A:Care-All, and Smart Coach. The exact values and standard error (in parenthesis) are displayed beside each dot. Intervention groups showed improvement, albeit statistically non-significant, in non-HDL-C and LDL-C as compared to the control. My A:Care-All displayed enhanced HDL-C levels as compared to the control (ANCOVA analysis, *p* = 0.091).

Clinical assessment of the participant's blood lipids was documented at baseline and week 12 to evaluate the influence of interventions on these parameters. Compared to the control, the intervention groups displayed marked improvement in non-HDL-C levels [% change (SE): My A:Care-All −5.5% (3.2), *p* = 0.213; Smart Coach −4.3% (3.7), *p* = 0.150; No-App −1.8% (3.7)] and a trend towards improvement in LDL-C levels [% change (SE): My A:Care-All −1.2% (4.0), *p* = 0.257; Smart Coach −2.1% (4.2), *p* = 0.111; No-App 1.3% (4.4)]. Additionally, the My A:Care-All group displayed a trend toward improvement in HDL-C levels (mean % [SE] change: 10.6 [2.9] vs. 2.6 [3.1]; *p* = 0.091) and TG (mean % change [SE]: −1.9 [6.1] vs. −0.5 [7.4]; *p* = 0.284). Other lipid parameters, including TC, LDL-C, and non-HDL-C, showed improvement in both intervention groups compared to the control; however, these changes did not reach statistical significance (Figure 3).

### 3.4 Secondary outcome: effectiveness of interventions on perception of medications

#### 3.4.1 Perception of medications in general

**Figure 4:**
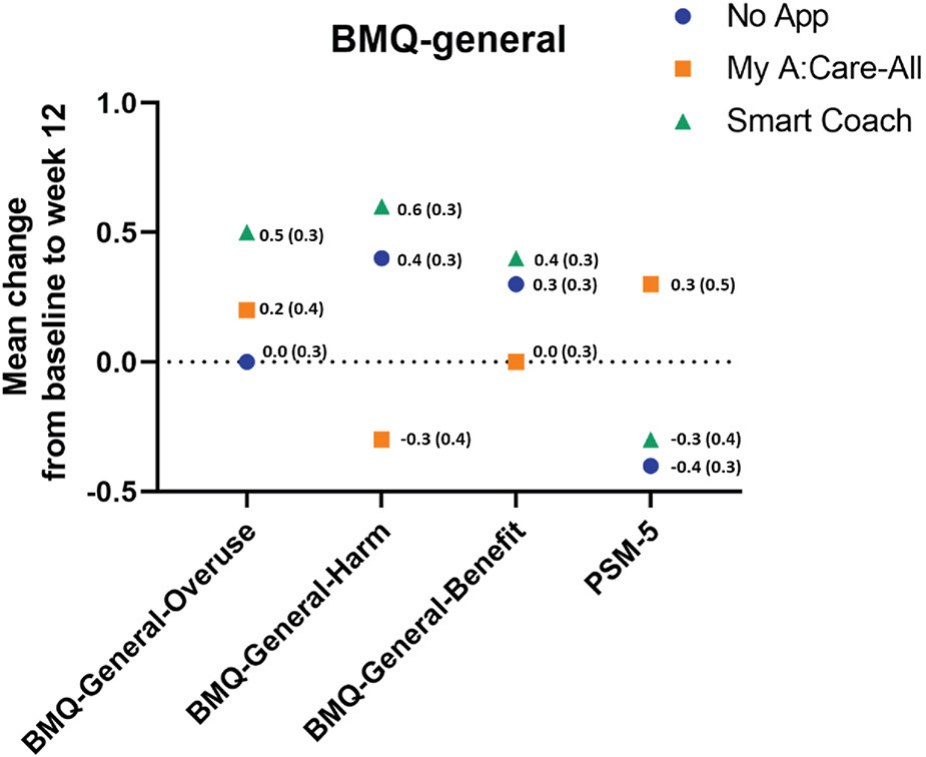
Perception to medication in general among study groups. The superimposed scatter plot depicts the changes in scores [mean change (SE)] for BMQ-General-12 and PSM-5 from baseline to week 12. Higher scores in the BMQ-General-12 and PSM indicate better perception to medicines and greater perceived sensitivity to potential adverse effects of medicines, respectively.

Patient's general perception of medications and their concerns about side effects were measured using BMQ-General-12 and PSM-5, respectively. At the beginning of the study, participants had a neutral to slightly positive perception of medication usage in general and a slightly negative perception of potential adverse effects. Patient's perception of medication did not change significantly during the study duration, either within the study groups or in between the control and App intervention arms (Figure 4 and Supplementary Table S4).

#### 3.4.2 Perception of medications specific to lipid-lowering therapy

**Figure 5:**
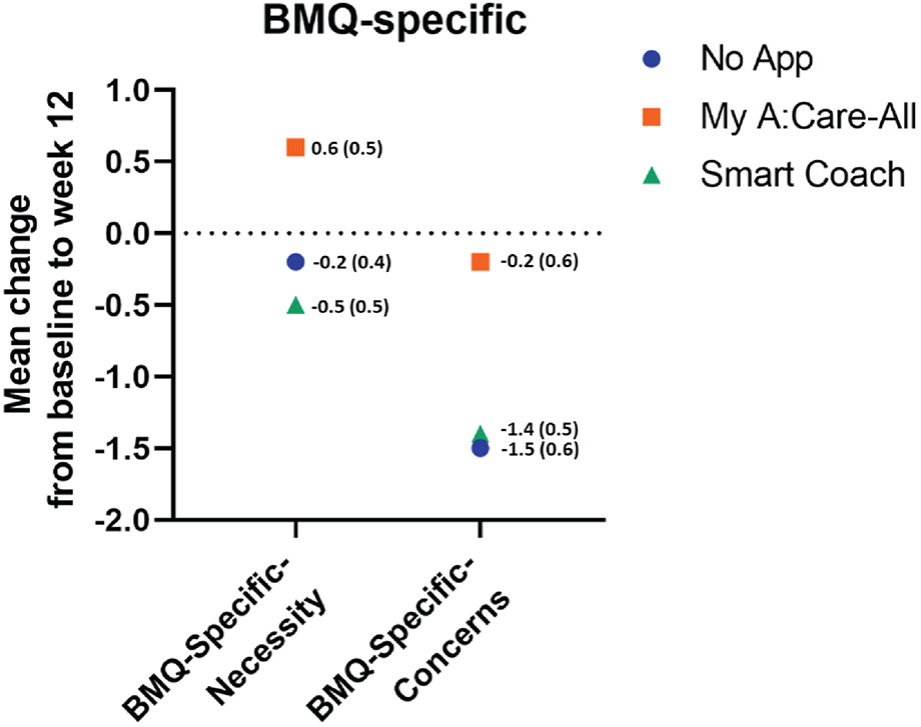
Perception specific to lipid-lowering medications among study groups. The superimposed scatter plot depicts the changes in scores [mean change (SE)] of BMQ-Specific-Necessity and BMQ-Specific-Concern. Higher score in the BMQ-Specific-Necessity and the BMQ-Specific-Concerns represent stronger perceptions of personal need for medicines and stronger concerns about the potential negative effects of the medicines, respectively.

Patient's specific perceptions of lipid-lowering agents were measured using the BMQ-S11 Plural questionnaire. At the baseline, patients had a positive perception of the need for the medicines and were relatively neutral toward potential negative effects. During the study, the BMQ-Specific-Necessity scores increased in the My A:Care-All group as compared to the control, but the change was not statistically significant (Mean [SD] 0.6 [3.7] vs. −0.2 [2.7]; *p*-value = 0.759). The BMQ-Specific-concern uniformly decreased across all study groups with no statistical difference among the groups (Figure 5 and Supplementary Table S5).

Furthermore, the MAIN Screen was used to determine the barriers and support needed for the participants' medicine adherence. At the end of the study, a higher proportion of participants reported no difficulty in using their medicines, higher support in using medicines, and higher availability of information and education across all the study groups. However, no significant difference was found between the groups in week 12 scores of MAIN (Supplementary Table S5).

### 3.5 Exploratory outcome: correlation between adherence to lipid-lowering therapy and mobile applications usage

As exploratory objectives, we tried to decipher the relationship between adherence to anti-lipidemic agents and mobile application usage. For these results, data from the My A:Care- Complete group was analyzed since the rest of the participants in the My A:Care-All did not receive motivational messages. We analyzed the cube (for engagement with motivational messages) and the battery status (for engagement with pill reminder) in the My A:Care- Complete vs. Smart Coach groups and also examined if there was any correlation between these two App features with MARS-5VA scores among the two study groups.

#### 3.5.1 Engagement with motivational messages

**Figure 6:**
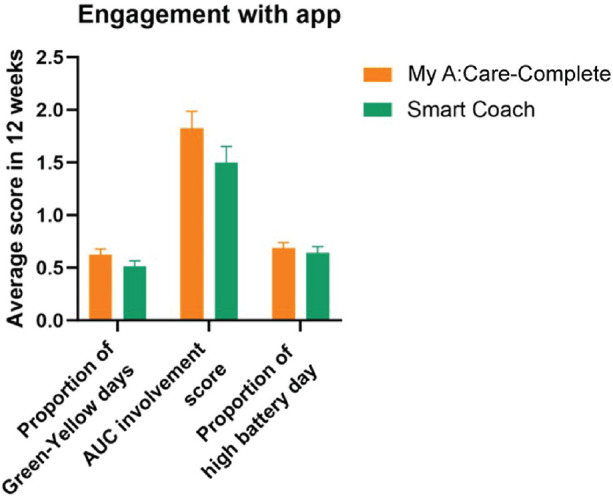
Patient engagement with the My A:Care and Smart Coach app. In both apps engagement with motivational features and messages is deciphered by cube status (Green: high; Yellow: low; Red: No engagement). The average engagement of the patients to this function is measured by AUC score. Similarly, battery status represents engagement with the reminder feature of the app. Error bars represent standard error (SE).

Participants engaged with motivational messages on at least 50% of the observational days. The proportion of green-yellow days in the cube was similar among the two groups (*p*-value = 0.866). Cube status can range from green, yellow, or red depending on the level of engagement and is measured using an AUC score on a scale of 1–3, with a higher score ascribed to a green cube. Both My A:Care- Complete and Smart Coach groups had similar AUC scores [mean (SD): 1.518 (1.282) vs. 1.501 (1.071); *p* = 0.891] (Figure 6).

#### 3.5.2 Engagement with pill reminder

Overall, engagement with pill reminders was found to be better as compared to motivational messages. Participants had high battery status on 64.3%–70.6% of observational days. The battery status was similar in My A:Care-Complete and Smart Coach groups [mean (SD): 0.706 (0.380) and 0.643 (0.413), respectively] (Figure 6).

#### 3.5.3 Correlation between medication adherence and App usage

The correlation between changes from baseline to week 12 in MARS-5VA scores and engagement with the mobile application was probed to understand the influence of app usage on improving adherence. The My A:Care-Complete group displayed a positive correlation between App usage and the change in MARS 5VA part 1 score (correlation coefficient ranging from 0.19 to 0.37). On the other hand, the usage of Smart Coach showed a weak negative correlation with the MARS 5VA part 1 score modifications (ranging from 0.10 to 0.19). It was perhaps due to outlier values in 7 participants, which negated the values of the whole group. Nevertheless, both these App usages did not yield any statistically significant correlations with changes in MARS-5VA part 1 scores at week 12 (Table 2).

**Table 2:** Correlation between improvement in medication adherence and engagement with the My A:Care and Smart Coach App.

App engagement variables	My A:Care-Complete (*N* = 16) Pearson's r (*p*-value)	Smart Coach (*N* = 49) Pearson's r (*p*-value)
Change in MARS-5VA part 1 score by		
Proportion of green-yellow day	0.37 (0.163)	-0.10 (0.492)
AUC involvement score	0.37 (0.158)	−0.10 (0.480)
Proportion of high battery day	0.19 (0.482)	−0.19 (0.201)
Change in MARS-5VA part 2 score		
Proportion of green-yellow day	0.25 (0.358)	0.05 (0.717)
AUC involvement score	0.22 (0.417)	0.05 (0.726)
Proportion of high battery day	0.04 (0.870)	0.29 (0.042)

Correlation between changes in MARS score and engagement with mobile applications.

The correlation between App usage and the change in MARS 5VA part 2 score in the My A:Care-Complete and Smart Coach groups was positive, ranging between 0.04 to 0.25 and 0.05 to 0.29. The correlation between high battery status and MARS 5VA part 2 scores in the Smart Coach group was statistically significant (Pearson's *r*: 0.29, *p* = 0.042) (Table 2).

## 4 Discussion

This pilot study evaluated the potential of the My A:Care and Smart Coach applications to improve medication adherence in patients newly diagnosed with dyslipidemia and on stable lipid-lowering therapy. Although no overall improvement in adherence was observed within any group from baseline, the intervention groups demonstrated greater persistence with therapy compared to the control group.

As is commonly observed with treatments for chronic and asymptomatic conditions, adherence to lipid-lowering therapy is highest at initiation but declines over time, with a significant proportion of patients discontinuing treatment within the first year (9–11). However, we did not observe a lowering in medication adherence during the 12-week study period in either of the two intervention groups as assessed by MARS-5VA part 1 scores. In comparison to the intervention groups, patients in the No App group displayed a significant decline in adherence. This demonstrates the utility of the two mobile applications in assisting patients to persist with their treatment regimen. We did not find any significant differences in MARS-5VA part 2 scores between the control and intervention groups. This might be partly because patients were relatively newly initiated to the therapy (9 months to 1 month before study commencement) and were required to take just one medication per day as per their treatment regimen. As an exploratory observation, during group comparison between My A:Care app with SMART coach for MARS-5VA part 1, we noted that if a non-inferiority margin of 0.25 had been pre-specified, the observed effect estimate of 0.02 with a 95% confidence interval of (–0.24, 0.19) would suggest that non-inferiority could potentially have been demonstrated within this margin (SupplementaryTable S3).

Enhanced persistence to anti-lipidemic agents results in an improvement in serum lipid parameters as observed in the intervention groups in comparison to the control. Both the intervention groups displayed improved levels of TC, LDL-C, and non-HDL-C. Furthermore, enhancement in HDL-C levels in the My A:Care-All group trended towards significance when compared to No App. These results are consistent with previous studies reporting the usefulness of mobile applications in improving adherence and leading to better regulation of lipid parameters (10, 33, 34). However, improvements in specific serum lipids were not consistent among different studies (33).

Both My A:Care and Smart Coach applications did not significantly impact patients' perception towards medication in general or specific to lipid-lowering agents, possibly due to a greater number of co-morbidities in the intervention groups. This could have resulted in higher use of medications, causing heightened concern-beliefs. The unexpected application malfunction in the My A:Care group, resulting in all but 16 patients not receiving the motivational messages, was a major challenge in the study. We performed extensive sensitivity analyses to minimize the bias arising from the uneven distribution of baseline characteristics in the three study groups. Another limitation was the use of self-reporting scales to measure adherence, as self-report scales are prone to reporting bias. This was a single-centre study involving a homogenous population, which could impact the generalizability of the results and must be followed up with larger multicenter studies. Despite these limitations, this proof-of-concept study offers valuable insights into the capabilities of behavioral model-based mHealth applications.

A recent review of 23 studies examined the impact of various mHealth applications on medication adherence in cardiovascular disease. Interventions ranged from simple text messaging to multifaceted approaches involving medication reconciliation, patient education, and tailored content. Of the studies reviewed, 17 reported improved medication adherence, while 6 showed limited or no significant impact (35). Another meta-analysis of RCTs evaluating digital interventions for cardiovascular risk factor modification reported improvements in health behaviors, including medication adherence, though it did not demonstrate benefits for clinical outcomes (18). Other studies have similarly demonstrated improvements, though the magnitude of effect was often modest, and the relationship between intervention and outcome was not always clearly established (36, 37). Consistent with these findings, our results indicated a modest but positive effect of both the My A:Care and Smart Coach applications on adherence to lipid-lowering therapy. These preliminary outcomes support the need for future larger and long-term studies to more rigorously evaluate the efficacy of these applications.

## 5 Conclusion

This study was designed as a proof-of-concept study to explore the impact of My A:Care and Smart Coach applications on adherence to the lipid-lowering therapy in subjects with dyslipidemia. The primary endpoint of adherence was positive, and potential benefits of the My A:Care app in lipid control were observed. These early signals of the effect of My A:Care app in improving adherence and enhancing lipid control warrant future studies with longer follow-up periods to conclusively assess the benefits. Future research could also focus on a more layered approach where healthcare providers are trained and equipped with tools to gauge non-adherence objectively in their patients and provide tailored support to manage non-adherence through the mHealth applications.

## Data Availability

The original contributions presented in the study are included in the article/Supplementary Material, further inquiries can be directed to the corresponding author.
