# Supplementary material for: Evaluating the effect of mobile applications “My A:Care” and “Smart Coach” on adherence to lipid-lowering treatment in patients with dyslipidemia: a prospective, randomized, open-label clinical study
Source: Front Digit Health. 2025 Jul 7;7:1502990. doi: 10.3389/fdgth.2025.1502990 (PMC12277276; doi:10.3389/fdgth.2025.1502990)
Supplement: Supplementary file 1 [file Datasheet1.docx]

# **S1. Supplementary Methods**

## **S1.1.** **A brief description about My A:care application**

My a:care is a novel mHealth application designed to address medication non-adherence using established behavioral methods. My a:Care associates adherence with self-care and helps patients take small, manageable steps while rewarding positive actions to facilitate lasting behavioral changes. The application provides motivational messages/challenges, health insights, and pill reminders to encourage adherence.

The application has two important components – 1) motivational challenges/messages and 2) medication reminders. The objective of this app is to work on the Motivation pillar of the COM-B model [1].

**Motivational challenge**

Patients are prompted several times a week to either complete a light motivational challenge or to read motivational texts. Following is an example of one such motivational challenge:

“Taking your medication on time helps your body get used to it and enhances the efficiency of the medication. Let’s make taking it fun :) How? By thinking of it as virtual candies that will bring all the sweetness your body needs to heal in the long run. This will trick your brain and help you get a better health, just by changing perspective!”

The patient is prompted to choose between the following responses:

- Yes, I want my candies!
- No, not really thanks

**Pill reminders**

Pill reminders work like usual medication reminders used in other mHealth applications. The medication details, frequency and dosage are filled at the time of initiation. Each reminder is pushed to the patient at the time of the medication intake as pre-scheduled in the application.

The medication battery aims at visually representing the amount of drug in the patient’s body. It permanently decreases until the battery becomes empty. When the patients takes his/her medication, the battery gets fully charged again.

**Cube status**

The level of motivation of the patient to self-care is represented with a green cube. The color of the cube will evolve between 4 status (green, yellow, orange, and red) according to the interactions with the app. If the patient stops interacting with the app, the color of the cube will evolve towards red. Committing to have a good behavior pushed the colors progressively back to green. Collecting green cubes in a row unlocks virtual rewards.


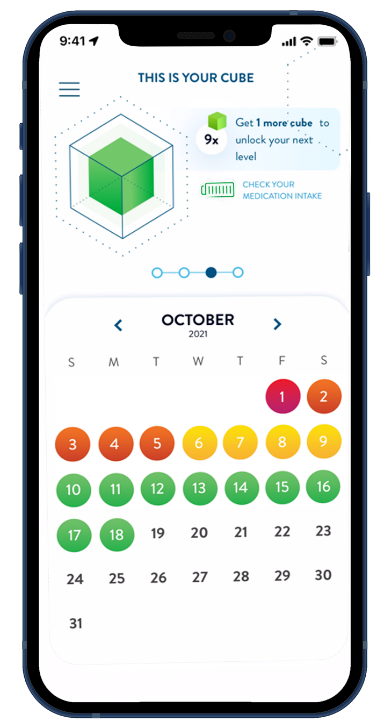


Suppl Figure S1: My A:care application interface showing the cube status.

## **S1.2. A brief description about** **My A:Care Smart Coach application**

My A:Care Smart Coach is an advanced version of the My A:Care application running on the same core engine with the same App interface. Similar to My A:care application, the Smart coach is based on behavioral methods to encourage self-care in patients. The interface of the application is same. The application includes both the main components of the My A:Care app, motivational challenges and medication reminders. The major difference is the use of SPUR (Social, Psychological, Usage, Rational) framework-enabled tailored treatment plans to patients. The SPUR questionnaire is administered at the time of app initiation for analysis. The SPUR questionnaire is a holistic profiling tool to quantify patients’ behavioral risks and the potential risk drivers [2]. Further, the SPUR results are assessed in combination with the patient’s clinical and sociodemographic information using d.Tells^TM^ algorithm [3] to determine the frequency, type, and content of the messages to be delivered to each individual. Thus, the motivational messages received by the app users are personalized based on individual risk assessment and risk drivers. The algorithm also recommends optimal interaction strategies to healthcare professionals for supporting each patient.

## **Supplementary Table 1: Features of My a:Care and SMART Coach applications:**

| **S.No.** | **App Feature** | **My a:Care app** | **SMART Coach app** |
| --- | --- | --- | --- |
| 1 | Adherence strategy used in the app | Reminder, behavioral, educational | Reminder, behavioral, educational |
| 2 | HCP involvement in development | Yes | Yes |
| 3 | Evidence-based | Yes | Yes |
| 4 | Behavioral model | COM-B, motivational messaging, behavioral nudges | COM-B, motivational messaging, behavioral nudges |
| 5 | Reminder based on alarm or "push-notification" or "SMS" | Push notification | Push notification |
| 6 | Tailored messaging based on barriers to adherence | No | Yes (SPUR tool) |
| 7 | Personal tracking | Yes | Yes |
| 8 | External monitoring | No | No |
| 9 | Gamification | Yes | Yes |
| 10 | Tracking other health metrics | No | No |
| 11 | Pharmacy information | No | No |
| 12 | Important contacts (pharmacis, emergency, family) | No | No |
| 13 | Refill reminder | Yes | Yes |
| 14 | Picture of the medication | Yes | Yes |
| 15 | Record medication and adherence information | Yes* patient-reported | Yes* patient-reported |
| 16 | Export/email medication and adherence information to HCPs and others | Yes | Yes |
| 17 | Appointment reminder | Yes | Yes |
| 18 | Barcode scanner to automatically input medicaiton information | No | No |
| 19 | Works/integratable with wearables | No | No |
| 20 | Available at both Google and Apple store | Yes | Yes |
| 21 | Languages available | Yes – but in the study, only Thai was used | Yes – but in the study, only Thai was used |

## **S1.3. Training details for the intervention groups**

As per the protocol, participants randomized to the mobile application groups were provided with a training session at Visit 1 (Screening/Baseline Visit – Day -14 to Day 1). The site staff helped the participants to download the allocated mobile applications through their respective smartphone application store, for example, the iTunes app store for smartphones that operate with iOS (e.g., iPhones) and the Google Play Store for smartphones that operate with Android system (e.g., Samsung, HTC, Huawei and Sony).

The site staff also demonstrated to the subject how to input their current list of medications into the application and respective indications by searching in the app’s database. Next, the site staff demonstrated how to select the appropriate dosage frequency (e.g., once daily) as well as how to set the time that the medication is usually taken (e.g., 08:00 AM). The site staff helped the subjects to enter the indicated dyslipidemia medications prescribed by their physicians and demonstrated how to edit or delete a medication if the medication dosage or timing is changed or suspended by the treating physician. After entering the medications in the mobile application, the site staff pointed out that every day, at the scheduled time, a reminder will pop-up on the smartphone screen, alerting the patient that it is time to take their medication.

# Supplementary Table 2: Adherence to Lipid-Lowering Therapy as assessed by MARS-5VA (Part 1and Part 2)

|  |  |  | No App (N=49) | My A:Care-Complete (N=16) | My A:Care-All (N=49) | Smart Coach (N=49) |
| --- | --- | --- | --- | --- | --- | --- |
|  | **Baseline** | | | | | |
| MARS-5VA Part 1 | Mean (SD) |  | 19.0 (0.6) | 18.9 (0.3) | 18.9 (0.6) | 18.9 (0.7) |
|  | Min/Max |  | 18 / 21 | 18 / 19 | 17 / 21 | 17 / 21 |
| MARS-5VA Part 2 | Mean (SD) |  | 85.1 (12.6) | 90.0 (15.9) | 88.0 (14.6) | 86.3 (15.5) |
|  | Min/Max |  | 50 / 100 | 50 / 100 | 50 / 100 | 40 / 100 |
|  | **Week 12** | | | | | |
| MARS-5VA Part 1 | Mean (SD) |  | 18.7 (0.8) | 18.8 (0.4) | 18.9 (0.3) | 18.9 (0.3) |
|  | Min/Max |  | 15 / 19 | 18 / 19 | 18 / 19 | 18 / 19 |
| MARS-5VA Part 2 | Mean (SD) |  | 95.1 (7.9) | 96.3 (6.2) | 95.9 (6.7) | 96.9 (6.5) |
|  | Min/Max |  | 70 / 100 | 80 / 100 | 70 / 100 | 80 / 100 |
|  | **Changes from Baseline to Week 12** | | | | | |
| MARS-5VA Part 1 | Mean (SD) |  | -0.3 (0.9) | -0.1 (0.6) | 0.0 (0.7) | 0.0 (0.7) |
|  | Min/Max |  | -4 / 1 | -1 / 1 | -2 / 2 | -2 / 2 |
| MARS-5VA Part 2 | Mean (SD) |  | 10.0 (14.6) | 6.3 (18.2) | 8.0 (15.0) | 10.6 (14.5) |
|  | Min/Max |  | -20 / 50 | -10 / 50 | -10 / 50 | -20 / 60 |

Abbreviations: MARS-5VA = Medication Adherence Report Scale with Visual Analogue Scale; Max = Maximum; Min = Minimum; SD = Standard Deviation.

Note 1: MARS-5VA Part 1 score ranges from 5 to 25 with high score indicative of high adherence to lipid-lowering medications.

Note 2: MARS-5VA Part 2 Week and Month scores are reported as percentage from 0 to 100 where 0 = no medicine used and 100 = all medicine used.

# Supplementary Table 3: Adherenec to lipid lowering therapy group comparisons

|  | Statistics | No App (N=49) vs. My A:Care-Complete (N=16) | No App (N=49) vs. Smart Coach (N=49) | No App (N=49) vs. My A:Care-All (N=49) | My A:Care-Complete (N=16) vs. Smart Coach (N=49) | My A:Care-All (N=49) vs. Smart Coach (N=49) |
| --- | --- | --- | --- | --- | --- | --- |
| MARS-5VA Part 1 |  |  |  |  |  |  |
| ANCOVA | Mean Difference (95% CI) | -0.13 (-0.44, 0.18) | -0.23 (-0.45, -0.02) | -0.21 (-0.43, 0.01) | -0.11 (-0.41, 0.20) | -0.02 (-0.24, 0.19) |
|  | Standard Error | 0.16 | 0.11 | 0.11 | 0.16 | 0.11 |
|  | p-value | 0.415 | 0.035 | 0.056 | 0.500 | 0.837 |
|  |  |  |  |  |  |  |
| Wilcoxon’s Rank-Sum | Z score | 0.56 | -1.68 | -1.45 | -0.69 | -0.37 |
|  | p-value | 0.571 | 0.093 | 0.147 | 0.485 | 0.707 |
|  |  |  |  |  |  |  |
| MARS-5VA Part 2 |  |  |  |  |  |  |
| ANCOVA | Mean Difference (95% CI) | -0.48 (-4.44, 3.47) | -1.52 (-4.28, 1.25) | -0.14 (-2.90, 2.62) | -1.03 (-4.98, 2.91) | -1.38 (-4.14, 1.38) |
|  | Standard Error | 2.00 | 1.40 | 1.40 | 2.00 | 1.39 |
|  | p-value | 0.809 | 0.280 | 0.921 | 0.605 | 0.324 |
|  |  |  |  |  |  |  |
| Wilcoxon’s Rank-Sum | Z score | -1.46 | 0.02 | 1.14 | -1.70 | -1.22 |
|  | p-value | 0.143 | 0.979 | 0.252 | 0.087 | 0.219 |
|  |  |  |  |  |  |  |
| Month |  |  |  |  |  |  |
| ANCOVA | Mean Difference (95% CI) | -0.69 (-4.70, 3.32) | -1.72 (-4.53, 1.08) | -0.55 (-3.35, 2.25) | -1.03 (-5.04, 2.97) | -1.17 (-3.97, 1.62) |
|  | Standard Error | 2.03 | 1.42 | 1.42 | 2.03 | 1.41 |
|  | p-value | 0.735 | 0.227 | 0.700 | 0.611 | 0.408 |
|  |  |  |  |  |  |  |
| Wilcoxon’s Rank-Sum | Z score | -1.36 | -0.11 | 0.98 | -1.76 | -1.28 |
|  | p-value | 0.170 | 0.906 | 0.327 | 0.078 | 0.200 |

ANCOVA = Analysis of Covariance; CI = Confidence Interval; MARS-5VA = Medication Adherence Report Scale with Visual Analogue Scale.

# Supplementary Table 4: Effectiveness of interventions in changing medication perception in general to lipid-lowering therapy.

**2 (a): Change from baseline to week 12 in the perception of medication in general and specific to lipid-lowering therapy**

|  | No App (N=49) Mean (SD) | My A:Care-complete (N=16) Mean (SD) | My A:Care-All (N=49) Mean (SD) | Smart Coach (N=49) Mean (SD) |
| --- | --- | --- | --- | --- |
| BMQ-General-Overuse | 0.0 (2.3) | -0.3 (3.1) | 0.2 (2.7) | 0.5 (2.1) |
| BMQ-General-Harm | 0.4 (2.5) | -0.5 (3.1) | -0.3 (2.6) | 0.6 (2.2) |
| BMQ-General-Benefit | 0.3 (2.0) | -0.7 (2.9) | 0.0 (2.5) | 0.4 (1.8) |
| PSM-5 | -0.4 (2.5) | 1.2 (3.5) | 0.3 (3.4) | -0.3 (2.7) |
| BMQ-Specific-Necessity | -0.2 (2.7) | 0.7 (4.0) | 0.6 (3.7) | -0.5 (3.5) |
| BMQ-Specific-Concerns | -1.5 (4.4) | 2.4 (4.6) | -0.2 (4.2) | -1.4 (3.2) |

**2 (b): Group comparisons for change from baseline to week 12 in the perception of medication in general and specific to lipid-lowering therapy**

|  | Statistics | No App  vs. My A:Care-All | No App  vs. Smart Coach | Smart Coach  vs. My A:Care-All |
| --- | --- | --- | --- | --- |
| BMQ-General-Overuse | Mean Difference (95% CI) | -0.56 (-1.34, 0.23) | -0.64 (-1.42, 0.14) | -0.09 (-0.87, 0.69) |
|  | Standard Error | 0.4 | 0.4 | 0.39 |
|  | p-value | 0.163 | 0.107 | 0.825 |
| BMQ-General-Harm | Mean Difference (95% CI) | 0.10 (-0.71, 0.90) | -0.03 (-0.83, 0.77) | -0.13 (-0.94, 0.69) |
|  | Standard Error | 0.41 | 0.4 | 0.41 |
|  | p-value | 0.816 | 0.935 | 0.757 |
| BMQ-General-Benefit | Mean Difference (95% CI) | 0.18 (-0.55, 0.92) | 0.22 (-0.52, 0.96) | 0.04 (-0.70, 0.78) |
|  | Standard Error | 0.37 | 0.37 | 0.38 |
|  | p-value | 0.624 | 0.559 | 0.912 |
| PSM-5 | Mean Difference (95% CI) | -0.44 (-1.41, 0.52) | 0.00 (-0.97, 0.97) | 0.44 (-0.52, 1.41) |
|  | Standard Error | 0.49 | 0.49 | 0.49 |
|  | p-value | 0.367 | 0.999 | 0.364 |
| BMQ-Specific-Necessity | Mean Difference (95% CI) | -0.18 (-1.34, 0.98) | 0.80 (-0.36, 1.96) | 0.98 (-0.17, 2.12) |
|  | Standard Error | 0.58 | 0.58 | 0.58 |
|  | p-value | 0.759 | 0.173 | 0.094 |
| BMQ-Specific-Concerns | Mean Difference (95% CI) | -0.63 (-1.87, 0.62) | 0.20 (-1.00, 1.40) | 0.84 (-0.40, 2.08) |
|  | Standard Error | 0.63 | 0.61 | 0.63 |
|  | p-value | 0.32 | 0.741 | 0.181 |

# Supplementary Table 5: Perception specific to lipid-lowering therapy – BMQ-S11-Plural and MAIN Screen scores summary.

|  | Statistic | No App (N=49) | My A:Care-Complete (N=16) | My A:Care-All (N=49) | Smart Coach (N=49) |
| --- | --- | --- | --- | --- | --- |
|  | | | | | |
| **Baseline** |  |  |  |  |  |
| BMQ-Specific-Necessity | Mean (SD) | 18.8 (2.6) | 18.3 (3.2) | 17.7 (3.8) | 17.8 (3.0) |
|  | Median | 19.0 | 19.0 | 19.0 | 18.0 |
|  | Min/Max | 14 / 24 | 12 / 22 | 5 / 24 | 9 / 24 |
|  | | | | | |
| BMQ-Specific-Concerns | Mean (SD) | 17.6 (3.8) | 15.4 (3.9) | 16.5 (4.6) | 17.0 (3.4) |
|  | Median | 17.0 | 15.0 | 16.0 | 17.0 |
|  | Min/Max | 11 / 26 | 11 / 22 | 6 / 28 | 12 / 25 |
|  | | | | | |
| MAIN Q1 - Difficulty of med use | A1 – Percent % | 79.6% | 56.3% | 65.3% | 83.7% |
|  | A2 – Percent % | 8.2% | 12.5% | 14.3% | 4.1% |
|  | A3 – Percent % | 12.2% | 25.0% | 16.3% | 10.2% |
|  | A4 – Percent % | 0.0% | 6.3% | 4.1% | 2.0% |
|  | | | | | |
| MAIN Q2 - Support to take med | A1 – Percent % | 65.3% | 62.5% | 59.2% | 55.1% |
|  | A2 – Percent % | 14.3% | 0.0% | 8.2% | 8.2% |
|  | A3 – Percent % | 20.4% | 18.8% | 20.4% | 24.5% |
|  | A4 – Percent % | 0.0% | 18.8% | 12.2% | 12.2% |
|  | | | | | |
| MAIN Q3 - Info and Advice about med | A1 – Percent % | 75.5% | 81.3% | 71.4% | 63.3% |
|  | A2 – Percent % | 0.0% | 0.0% | 2.0% | 2.0% |
|  | A3 – Percent % | 24.5% | 18.8% | 26.5% | 34.7% |
|  | A4 – Percent % | 0.0% | 0.0% | 0.0% | 0.0% |
|  | | | | | |
| MAIN Q4 - Importance of med | A1 – Percent % | 0.0% | 0.0% | 0.0% | 0.0% |
|  | A2 – Percent % | 2.0% | 0.0% | 0.0% | 0.0% |
|  | A3 – Percent % | 51.0% | 56.3% | 44.9% | 63.3% |
|  | A4 – Percent % | 46.9% | 43.8% | 55.1% | 36.7% |
|  | | | | | |
| MAIN Q5 - Concern about med | A1 – Percent % | 34.7% | 56.3% | 42.9% | 36.7% |
|  | A2 – Percent % | 46.9% | 25.0% | 38.8% | 53.1% |
|  | A3 – Percent % | 16.3% | 18.8% | 16.3% | 6.1% |
|  | A4 – Percent % | 2.0% | 0.0% | 2.0% | 4.1% |
|  | | | | | |
|  |  |  |  |  |  |
| MAIN Q6 - Problem with med supply | A1 – Percent % | 81.6% | 81.3% | 79.6% | 81.6% |
|  | A2 – Percent % | 8.2% | 12.5% | 14.3% | 14.3% |
|  | A3 – Percent % | 10.2% | 6.3% | 6.1% | 4.1% |
|  | A4 – Percent % | 0.0% | 0.0% | 0.0% | 0.0% |
|  | | | | | |
| **Week 12** |  |  |  |  |  |
| BMQ-Specific-Necessity | Mean (SD) | 18.6 (2.4) | 18.9 (3.5) | 18.2 (3.1) | 17.3 (3.9) |
|  | Median | 19.0 | 19.5 | 18.0 | 18.0 |
|  | Min/Max | 14 / 25 | 12 / 25 | 12 / 25 | 9 / 25 |
|  | | | | | |
| BMQ-Specific-Concerns | Mean (SD) | 16.1 (3.3) | 17.8 (3.3) | 16.3 (4.0) | 15.7 (2.9) |
|  | Median | 15.0 | 17.5 | 16.0 | 16.0 |
|  | Min/Max | 11 / 28 | 13 / 24 | 8 / 26 | 7 / 25 |
|  | | | | | |
| MAIN Q1 - Difficulty of med use | A1 – Percent % | 87.8% | 75.0% | 79.6% | 87.8% |
|  | A2 – Percent % | 8.2% | 25.0% | 16.3% | 4.1% |
|  | A3 – Percent % | 4.1% | 0.0% | 4.1% | 8.2% |
|  | A4 – Percent % | 0.0% | 0.0% | 0.0% | 0.0% |
|  | | | | | |
| MAIN Q2 - Support to take med | A1 – Percent % | 71.4% | 68.8% | 81.6% | 83.7% |
|  | A2 – Percent % | 16.3% | 6.3% | 6.1% | 10.2% |
|  | A3 – Percent % | 8.2% | 18.8% | 10.2% | 4.1% |
|  | A4 – Percent % | 4.1% | 6.3% | 2.0% | 2.0% |
|  | | | | | |
| MAIN Q3 - Info and Advice about med | A1 – Percent % | 75.5% | 87.5% | 85.7% | 77.6% |
|  | A2 – Percent % | 8.2% | 6.3% | 4.1% | 2.0% |
|  | A3 – Percent % | 16.3% | 6.3% | 10.2% | 20.4% |
|  | A4 – Percent % | 0.0% | 0.0% | 0.0% | 0.0% |
|  | | | | | |
| MAIN Q4 - Importance of med | A1 – Percent % | 0.0% | 0.0% | 0.0% | 0.0% |
|  | A2 – Percent % | 4.1% | 0.0% | 0.0% | 0.0% |
|  | A3 – Percent % | 36.7% | 56.3% | 49.0% | 55.1% |
|  | A4 – Percent % | 59.2% | 43.8% | 51.0% | 44.9% |
|  | | | | | |
| MAIN Q5 - Concern about med | A1 – Percent % | 46.9% | 31.3% | 34.7% | 34.7% |
|  | A2 – Percent % | 42.9% | 18.8% | 38.8% | 57.1% |
|  | A3 – Percent % | 8.2% | 43.8% | 22.4% | 8.2% |
|  | A4 – Percent % | 2.0% | 6.3% | 4.1% | 0.0% |
|  | | | | | |
| MAIN Q6 - Problem with med supply | A1 – Percent % | 91.8% | 93.8% | 89.8% | 87.8% |
|  | A2 – Percent % | 6.1% | 6.3% | 10.2% | 10.2% |
|  | A3 – Percent % | 2.0% | 0.0% | 0.0% | 2.0% |
|  | A4 – Percent % | 0.0% | 0.0% | 0.0% | 0.0% |
|  | | | | | |
| **Change from Baseline to Week 12** |  |  |  |  |  |
|  |  |  |  |  |  |
| BMQ-Specific-Necessity | Mean (SD) | -0.2 (2.7) | 0.7 (4.0) | 0.6 (3.7) | -0.5 (3.5) |
|  | Median | 0.0 | 0.0 | 0.0 | 0.0 |
|  | Min/Max | -6 / 6 | -4 / 13 | -8 / 13 | -11 / 8 |
|  | | | | | |
| BMQ-Specific-Concerns | Mean (SD) | -1.5 (4.4) | 2.4 (4.6) | -0.2 (4.2) | -1.4 (3.2) |
|  | Median | -2.0 | 3.0 | -1.0 | -1.0 |
|  | Min/Max | -12 / 12 | -8 / 9 | -11 / 9 | -7 / 6 |

Abbreviations: BMQ = Beliefs About Medicines Questionnaire; MAIN = Medicines Adherence And Information Needs; Max = Maximum; med = medications; Min = Minimum; SD = standard deviation.

Note 1: The BMQ-Specific-Necessity score ranges from 5 to 25 with higher scores representative of stronger perceptions of personal need for the medicines to maintain health now and in the future.

Note 2: The BMQ-Specific-Concerns score ranges from 6 to 30 with higher scores representative of stronger concerns about the potential negative effects of the medicines.

1. Christina Jackson, Â. L. E., Nick Barber, John Weinman (2014). Applying COM-B to medication adherence: A suggested framework for research and interventions. *The European Health Psychologist, 16*(1), 7-17.

2. Dolgin, K. (2020). The SPUR Model: A Framework for Considering Patient Behavior. *Patient Prefer Adherence, 14*, 97-105, doi:10.2147/PPA.S237778.

3. observia (2023). d.tells™. <https://observia-group.com/en/product/d-tells>.
